# Digitalis in Heart Diseases

**Published:** 1875-04

**Authors:** 


Digitalis in Heart Diseases.—*Prof. Wood.*—Digitalis, then, in man, by its action on the inhibitory apparatus, prolongs the period of diastole, thus giving time for the ventricles to fill up with more blood than usual, and also increases the muscular power of the heart, so that when it contracts a greater volume of blood is thrown with a greater force into the arterial system. Before we begin to apply these principles, remember also that the vascular system under the control of the vaso-motor nerves is probably kept in a state of contraction by the influence of digitalis.

Almost nothing but common sense is needed now to apply these facts to the treatment of heart diseases. If what has been said is true, digitalis ought to be useful when there is a deficiency of heart-power. Remember that it is not a rag that will stop a leak, and do not fall into the common error of expecting the drug to perform impossibilities. It cannot tighten a leaking valve. It cannot open and smooth down a contracted orifice. In other words, in valvular lesions it can only indirectly remedy the defects; and although often you will get the most surprising results from its use, yet in every case of valvular lesion there comes, sooner or later, a stage when digitalis is powerless. It is when the valves are healthy, and the cardiac failure is due simply to the weakness of the muscular walls that digitalis exerts its most wonderful powers. Nothing is more marvelous in clinical medicine than the relief you can sometimes rapidly afford in cases of simple dilatation of the heart.—*Canada Medical Record.*

